# Supplementary material for: Phytochemical Screening of Himatanthus sucuuba (Spruce) Woodson (Apocynaceae) Latex, In Vitro Cytotoxicity and Incision Wound Repair in Mice
Source: Plants (Basel). 2021 Oct 16;10(10):2197. doi: 10.3390/plants10102197 (PMC8541601; doi:10.3390/plants10102197)
Supplement: Supplementary file 1 [file plants-10-02197-s001.zip › plants-1417087-supplementary.pdf]

Supplementary materials

Phytochemical Screening of *Himatanthus sucuuba* (Spruce) Woodson (Apocynaceae) latex, in-vitro cytotoxicity and incision wound repair in mice

Oscar Herrera-Calderón [1], Lisbeth Lucia Calero-Armijos [1], Wilson Cardona-G [2], Angie Herrera-R [2], Gustavo Moreno [2], Majed A. Algarni [3], Mohammed Alqarni [4], Gaber El-Saber Batiha [5]

[1] Department of Pharmacology, Bromatology, Toxicology, Faculty of Pharmacy and Biochemistry, Universidad Nacional Mayor de San Marcos; oherreraca@unmsm.edu.pe (O.H-C); lisluc21@gmail.com (L.L.C-A.)

[2] Química de Plantas Colombianas, Faculty of Exact and Natural Sciences, Institute of Chemistry, University of Antioquia (UdeA), Calle 70 No. 52–21, Medellín AA 1226, Colombia, wilson.cardona1@udea.edu.co (W.C.-G), angie.herrerar@udea.edu.co (A.H-R.); gustavomorenoq@gmail.com (G.M)

[3] Department of Clinical Pharmacy, College of Pharmacy, Taif University, P.O. Box 11099, Taif 21944, Saudi Arabia; m.alqarni@tu.edu.sa (M.A.A);

[4] Department of Pharmaceutical Chemistry, College of Pharmacy, Taif University, P.O. Box 11099, Taif 21944, Saudi Arabia; m.alqarni@tu.edu.sa (M.A.);

[5] Department of Pharmacology and Therapeutics, Faculty of Veterinary Medicine, Damanhour University, Damanhour, Egypt. dr_gaber_batiha@vetmed.dmu.edu.eg (M.A.).

* Correspondence: oherreraca@unmsm.edu.pe; Tel.: (+51 956 550 510)

Supplementary figures

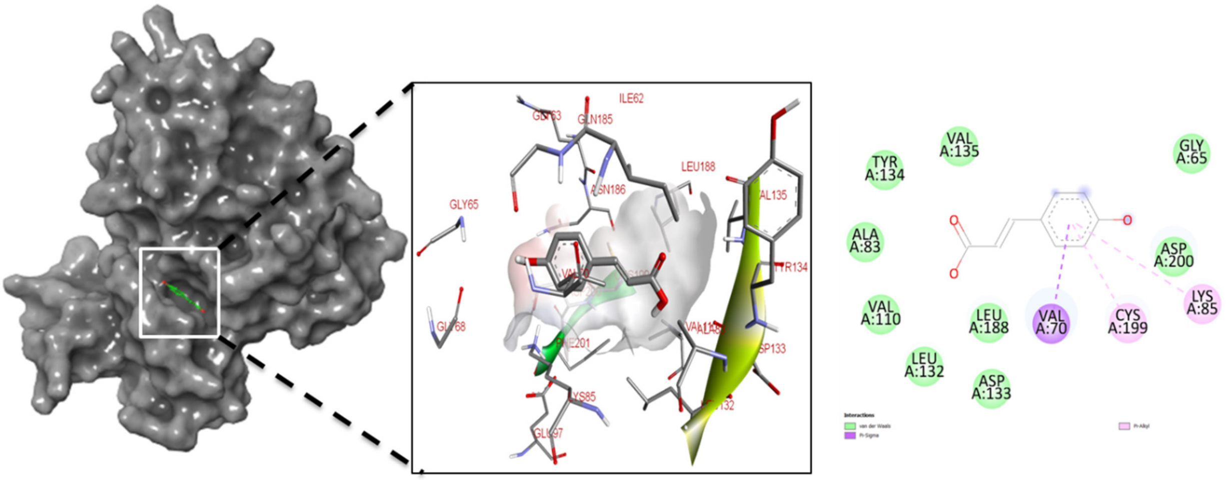

trans-4-Coumaric acid

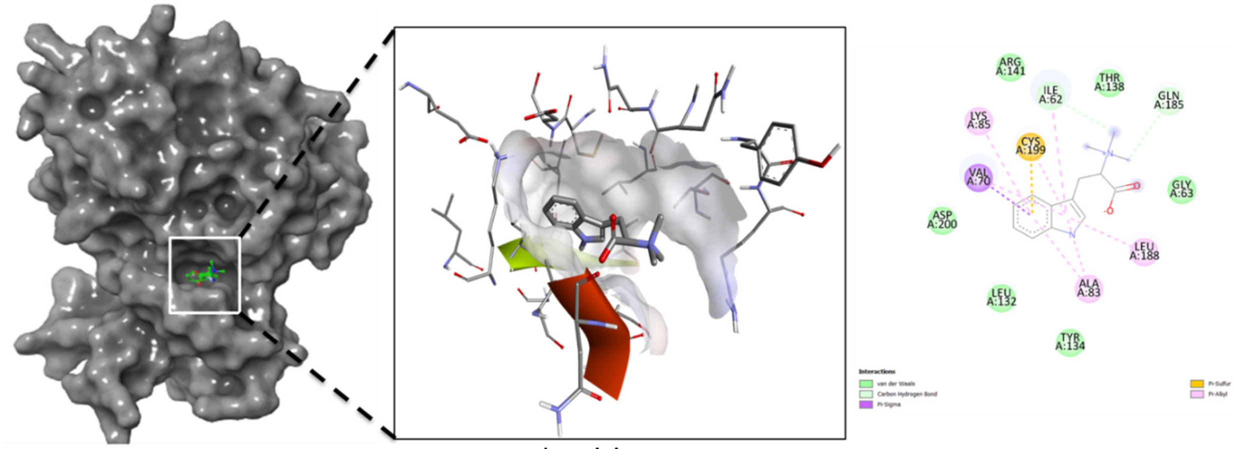

Lenticin

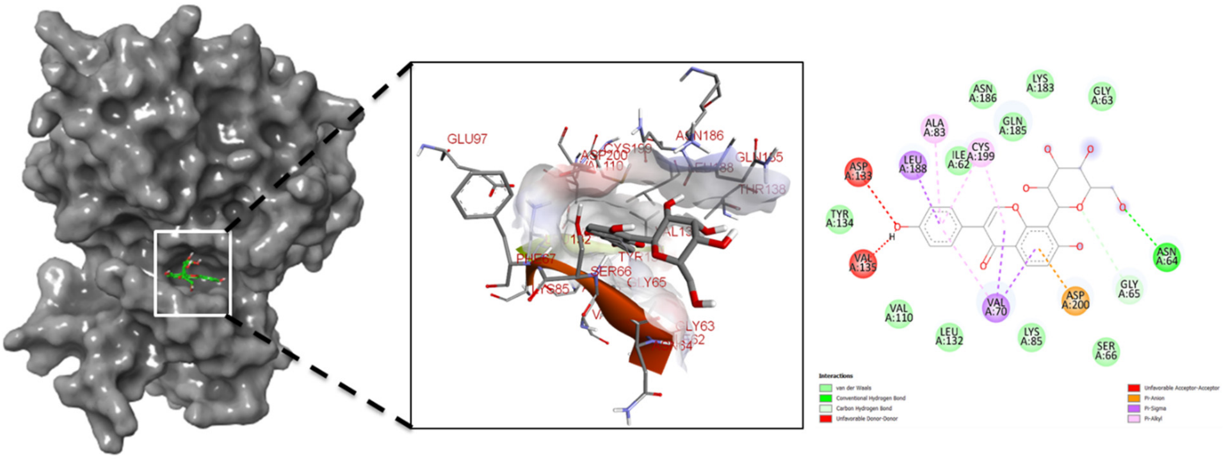

Puerarin

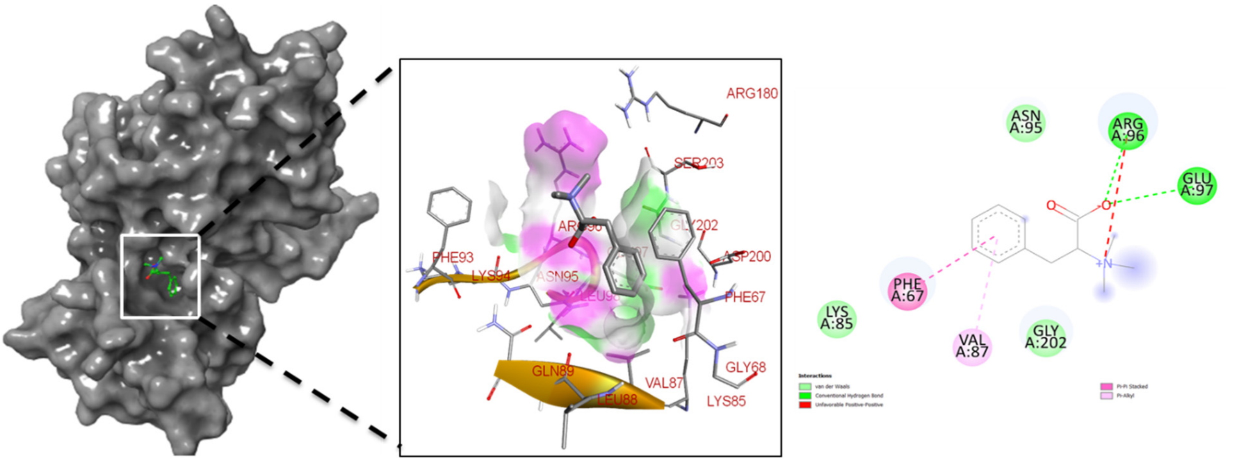

Phenylalanine betaine

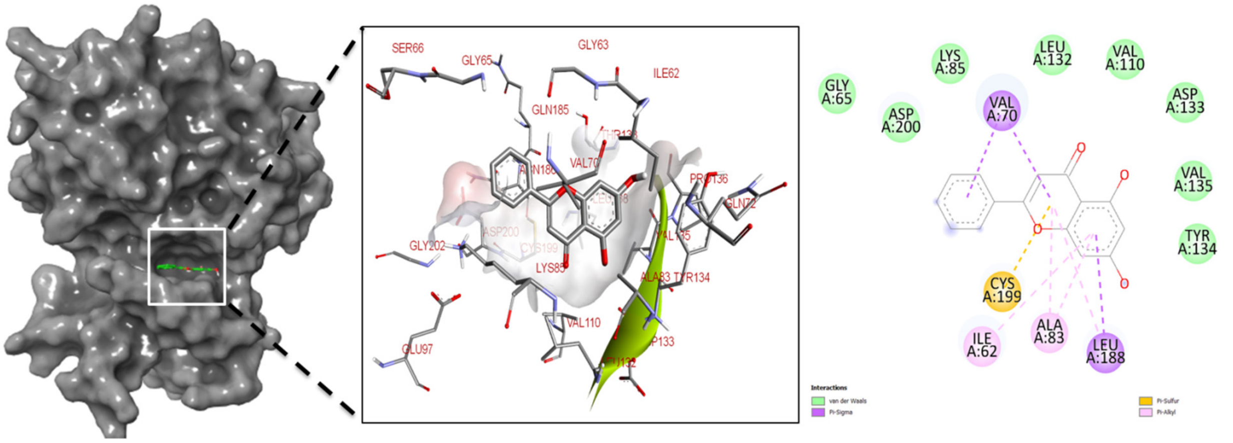

Chrysin

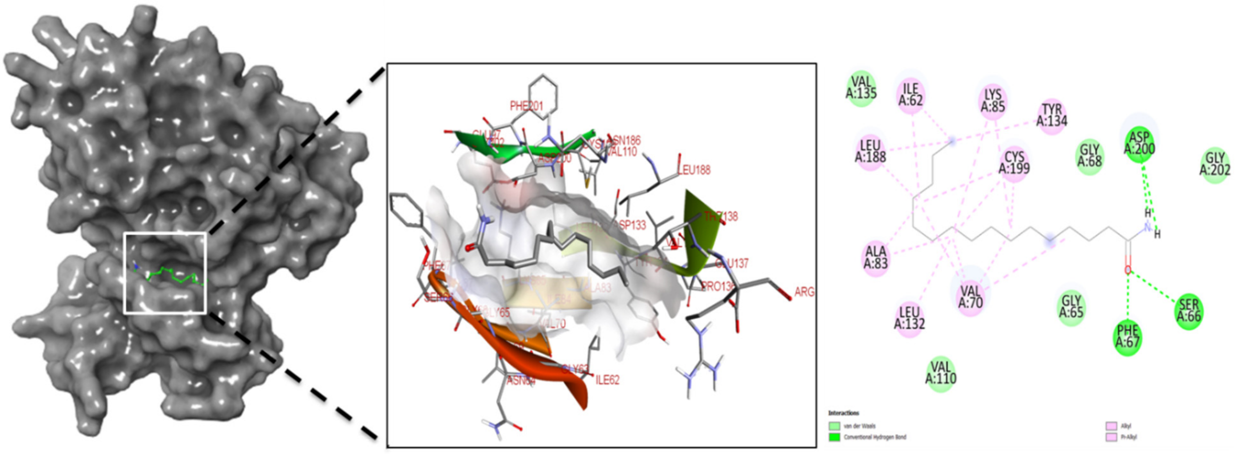

Palmitamide

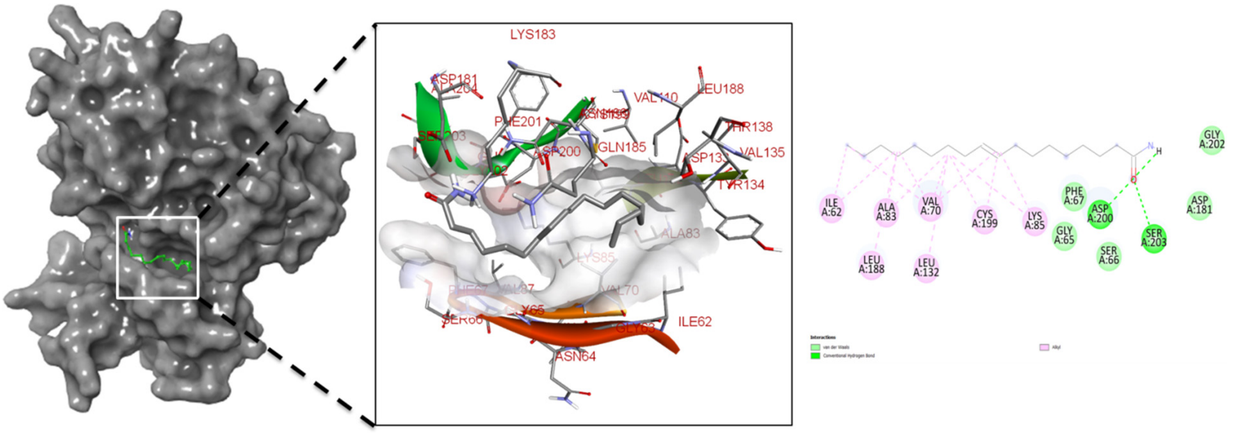

9-Octadecenamide

Figure S1. Molecular interactions studies of the phytochemical constituents of *H. sucuuba* latex with the Glycogen synthase kinase 3-β (PDB IDs: 1Q5K), surface view (Left panel) and 2D (Right panel) interactions.

Table S1. Representative images of SW480, SW620 and CHO-K1 cells 48 h after treatment with 1% DMSO (Magnification: 40x).

UNTREATED CELLS	CELLS WITH DMSO (1%)
**SW480** 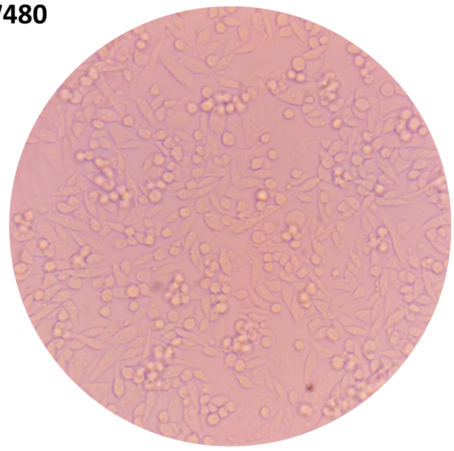	**SW480** 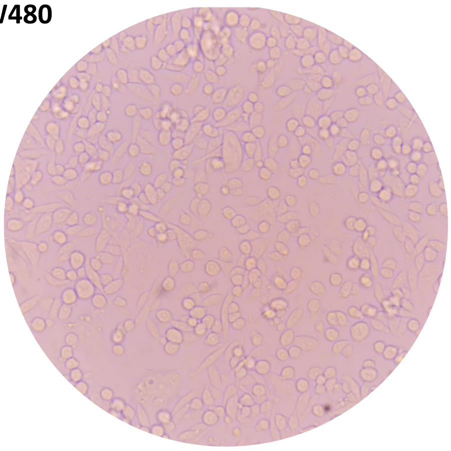
**SW620** 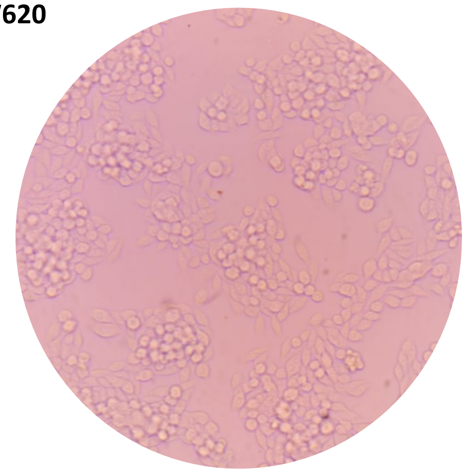	**SW620** 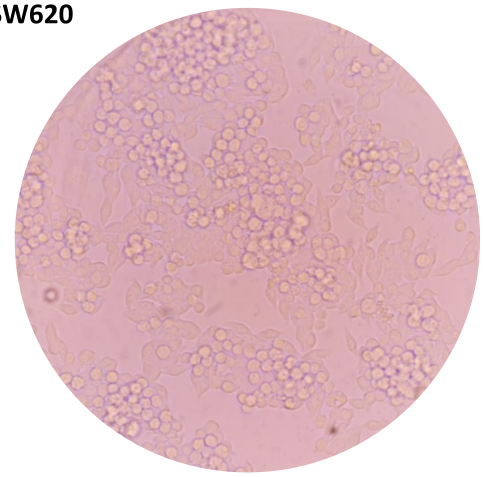
**CHO-K1** 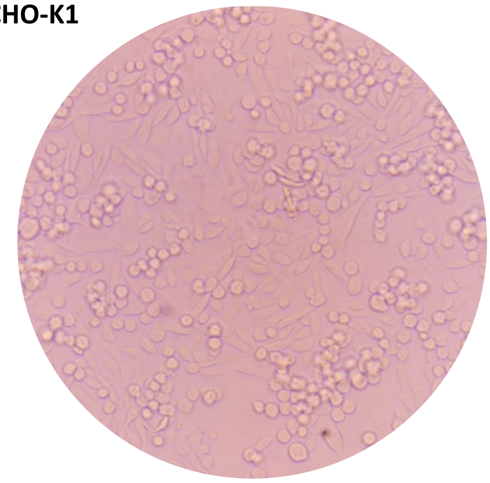	**CHO-K1** 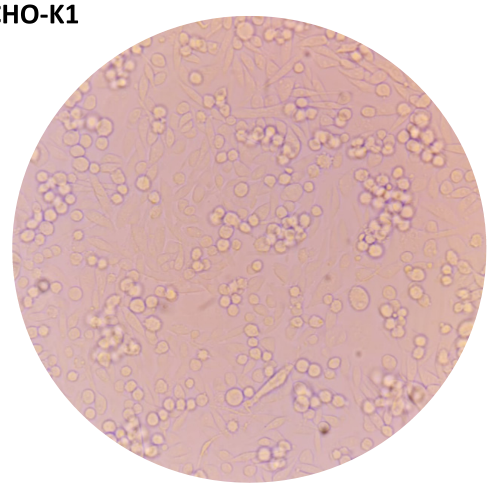
